# 16S amplicon-based microbiome biomapping of a commercial broiler hatchery

**DOI:** 10.1186/s42523-024-00334-3

**Published:** 2024-08-09

**Authors:** Michael J. Rothrock, Benjamin Zwirzitz, Walid G. Al Hakeem, Adelumola Oladeinde, Jean Y. Guard, Xiang Li

**Affiliations:** 1 Egg and Poultry Production Safety Research Unit, USDA-ARS, US National Poultry Research Center, 950 College Station Rd., Athens, GA USA; 2 Institute of Food Science, University of Natural Resources and Life Sciences, Vienna, Austria; 3 Oak Ridge Institute for Science and Education, US-DOE, Oak Ridge, Tennessee USA

**Keywords:** Microbiome, Microbiota, Biomapping, Hatchery

## Abstract

Hatcheries, where eggs from multiple breeder farms are incubated and hatched before being sent to different broiler farms, represent a nexus point in the commercial production of broilers in the United States. Considering all downstream microbial quality and safety aspects of broiler production (live production, processing, consumer use) can be potentially affected by the hatchery, a better understanding of microbial ecology within commercial hatcheries is essential. Therefore, a commercial broiler hatchery was biomapped using 16S rRNA amplicon-based microbiome analyses of four sample type categories (Air, Egg, Water, Facility) across five different places in the pre-hatch, hatch, and post-hatch areas. While distinct microbiota were found for each sample type category and hatchery area, microbial community analyses revealed that Egg microbiota trended towards clustering with the facility-related samples when moving from the prehatch to post-hatch areas, highlighting the potential effect of the hatchery environment in shaping the pre-harvest broiler-related microbiota. Prevalence analyses revealed 20 ASVs (Core20) present in the core microbiota of all sample types and areas, with each ASV possessing a unique distribution throughout the hatchery. Interestingly, three Enterobacteriaceae ASVs were in the Core20, including *Salmonella*. Subsequent analyses showed that *Salmonella,* while a minor prehatch and hatch Core20ASV, dominated the Enterobacteriaceae niche and total microbiota in the chick pad feces in the post-hatch area of the hatchery, and the presence of this *Salmonella* ASV in the post-hatch feces was associated with swabs of breakroom tables. These findings highlight the complexity of commercial hatchery microbiota, including identifying chick pad feces and breakroom tables as potentially important sampling or disinfection targets for hatchery managers to focus their *Salmonella* mitigation efforts to reduce loads entering live production farms.

**Supplementary Information:**

The online version contains supplementary material available at 10.1186/s42523-024-00334-3.

## Introduction

The vertically integrated poultry industry in the United States operates within a production pyramid structure, with pedigree or elite breeds positioned at the apex and broilers at the base. Broiler hatcheries occupy a central role between parent stock and their offspring [1]. This physical separation forms a potential barrier protecting the progeny from various pathogenic bacteria. Despite the clear separation between the breeders and their offspring, there are various factors related to egg and chick biology, as well as the hatchery operations and management, that can drive the development of poultry microbiota through live production and into the processing environments [2]. For example, previous studies have shown that core members of the gastrointestinal tract microbiota of the embryonic chicks in the hatchery can be identified in the poultry-associated core microbiota at later stages of development (live production, processing, final product) [3]. Additionally, a recent meta-analysis of preharvest poultry effects on the microbial risk of poultry meat has attributed nearly half (48.5%) of this risk to the hatchery environment [4].

Around 9.8 billion broiler chickens were hatched, raised, and processed last year in the United States alone [5]. Because of the intensive nature of the vertically integrated poultry production system, hatcheries can become vulnerable to zoonotic organisms that can come from various sources and spread to many downstream farms and processing facilities [1]. Foodborne bacterial pathogens such as *Salmonella*, *E. coli*, and various other Enterobacteriaceae can be located on or within the egg surface and on fomites such as egg trays and egg trolleys [6–8]. Infiltration through the eggshell/membrane into the egg contents can help these bacterial pathogens evade the extensive disinfection procedures used within commercial hatcheries [9]. While *Salmonella* and *E.coli* can be lethal to embryos during incubation [10], egg incubation conditions can allow these foodborne pathogens to proliferate, and chicks can still hatch from heavily contaminated eggs [7]. This leads to an increased risk of horizontal transmission of infectious agents not only between adjacent trays and other chick processing and storage equipment within the hatchery (resulting in potential foodborne pathogen reservoirs within the facility), but also between newly hatched chicks with relatively naive native gut microbiota [11].

Traditionally, microbial assessment through culturing is the gold standard for evaluating the presence and the control of foodborne bacterial pathogens in the hatchery. Several studies have investigated the presence of pathogenic bacteria such as *Salmonella*, *E. coli*, and *Mycoplasma* by culture-dependent methods; however, these measurements do not typically provide a comprehensive picture of the changes in the general microbial populations and how they may relate to the ecology of these pathogens [8, 11, 12]. 16S rRNA gene-based sequencing has emerged as an alternative, offering in-depth exploration of microbial communities within poultry facilities. Poultry houses and processing plants have been previously biomapped in various studies [13–15], highlighting shifting poultry-related microbiota in the different areas and stages of live production and processing. Additionally, *Pseudomonas*, *Enterobacteriaceae*, and *Weeksellaceae Chryseobacterium* have been identified as potential foodborne pathogen indicator organisms that have been isolated from all the samples and sampling locations in processing facilities [14].

Given its importance in influencing downstream pre-harvest and post-harvest poultry production, it is vital to biomap commercial hatchery facilities. This study characterizes the microbial communities found in different areas and sample types in a commercial broiler hatchery. Biomapping the different hatchery microbiota can potentially identify the role of hatcheries in shaping the gut microbiota in newly hatched broilers, as well as identifying areas or sample types within the hatchery for facility managers to focus foodborne pathogen mitigation strategies to minimize the flow of these pathogens to the live production farms.

## Materials and methods

### Facility description

**Fig. 1:**
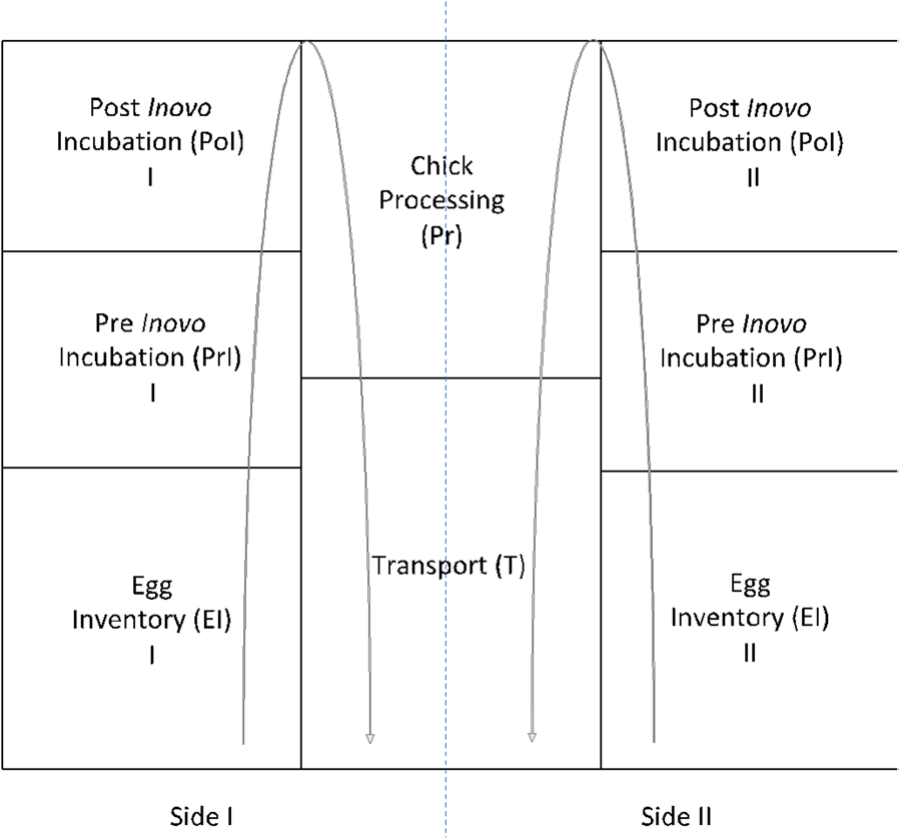
Schematic of the commercial hatchery facility sampled in this study. The facility was split into two mirrored halves (Side I and II), and the arrows represent the movement of the eggs through the facility

The overall layout of the commercial hatchery facility is shown in Fig. 1. The facility was divided into 2 halves that mirror one another (Side I and II). The facility is divided into 5 main areas:

1. Egg Inventory **(EI)** – Area where the eggs are delivered from the broiler breeder farms and stacked in racks for 1 to 3 days at 17 to 20 °C.
2. Pre-*in ovo* Incubation **(PrI)** – Area containing incubator rooms where the eggs are maintained at 37 to 38 °C up to 19 days.
3. Post*-in ovo* Incubation **(PoI)** – Area containing incubator rooms where eggs are placed after the *in ovo* immunizations and maintained at 37 to 38 °C for 36 to 48 h until hatch.
4. Chick Processing **(PR)** – Area where hatched chicks are mechanically separated from the eggshells, placed in racks, and a commercial live-attenuated *Salmonella* vaccine is administered.
5. Transport **(T)** – Area where newly hatched chicks are placed in stacking trays until being loaded on the transport trucks to the live production farms.

In this facility, the first three areas (EI, PrI, PoI) are on separate sides of the facility, but both sides of the facility use the same PR and T areas. For this study, eggs/chicks from two separate broiler breeder flocks (30 to 35 weeks of age) were followed from both halves of the hatchery areas on two separate production days.

### Hatchery sampling and sample processing

For the pre-hatch areas of the facility (EI, PrI, and PoI), egg, water, and air samples were collected using 3 M™ Sponge-Stick swabs pre-soaked with DE neutralizing buffer (Neogen, Inc., Lansing, MI). For egg samples, 25 eggs were randomly selected, and the entire shell surfaces were swabbed with the same sponge-stick. For air samples, the filter over the air intake (EI; 30 cm × 30 cm area), or multiple fan blades moving the air throughout the incubator units (PrI, PoI) were swabbed per flock replicate. For the water samples, the surfaces of the floor drains located throughout the areas were swabbed with a single sponge-stick for each area per flock replicate. While the air and water samples were taken as described above for the PR area, the egg-related sample consisted of filling a 1-gallon Ziploc bag with eggshells and fluff that were mechanically separated from the chicks. Additionally, the conveyor belt that removed the eggshells/fluff after separation from the chicks was also swabbed, swabbing the belt for 10 secs as the processing was occurring. For the T area, the egg-related samples were represented by the meconium/fecal samples from the chicks in the transport trays collected on fresh chick pads placed under the chicks for 30 min before being loaded onto the transport truck. The air and water T samples were taken from the transport truck to the live production farm prior to loading the chicks. The air samples (fan blades within the truck) were sampled as described above, and the water samples came from swabbing a 30 cm × 30 cm area of the underfloor water reservoir areas.

Facility-associated samples (FAC) were also taken. These included (1) sampling the inlet/outlet areas of rodent bait boxes (multiple boxes in each area were swabbed with a single sponge-stick, but new sponge-sticks were used in each area), (2) sampling the tables of the worker breakroom area, (3) sampling the faucets/sinks of the worker restrooms, and (4) sampling a 30 cm × 30 cm floor area of the highest foot traffic area in the facility (as identified by the facility manager). Additionally, 200 mL samples of (5) the incoming city water and (6) the outgoing wastewater were also collected in 250 mL Nalgene bottles. The breakdown of sample numbers by hatchery area and sample type can be found in Supplemental Table S1.

Once the samples were taken, the swabs were placed back into the provided sampling bags, and all samples (swabs, eggshells/fluff, feces, water) placed on ice until returned to the laboratory for further processing. The different types of samples were processed as follows:

1. Sponge-stick swabs. To prepare the swab samples for homogenization, the sponge-swabs were aseptically placed into filtered stomacher bags (Seward Laboratories Systems, Inc., West Sussex, UK), and 30 mL of 10 mmol L^−1^ phosphate-buffered saline (PBS) was added.
2. Eggshell/fluff. The collected sample was weighed, placed into the filtered stomacher bag, and then diluted 1:3 with 10 mmol L^−1^ PBS.
3. Chick pad feces. Three chick pads from each replicate were placed in 2-gallon Ziploc bag and 750 mL of buffered peptone water (BPW) was added. Afterwards, bags were shaken by hand for 2 min and incubated for 1 h at 37 °C. Thereafter, ~ 200 ml aliquots of chick pad rinsate were transferred to 250 mL Nalgene centrifuge bottles and centrifuged at 4,600 × g for 5 min. After centrifugation, the pelleted rinsate was resuspended in the residual BPW (~ 5 mL) in each bottle.
4. City water. 400 mL water was filtered through a 0.2-micron isopore membrane (Millipore Sigma). Following filtration, the filter was removed and placed in a stomacher bag with 10 mL BPW.
5. Wastewater. Two additional filtration steps were performed to remove debris. First, 200 mL wastewater was passed through two sheets of sterile cheesecloth and the filtrate was collected. Afterwards, the filtrate was filtered through a double layer of membrane filters (upper filter; 1.2- micron cellulose membrane, bottom filter; 0.2-micron isopore membrane). After filtration, the “double layered filter” was transferred to a stomacher bag containing 10 mL BPW. This process was completed twice per samples to process a total of 400 mL of wastewater.

Regardless of sample type, all samples were then homogenized for 30 s at 230 rpm in a Seward Stomacher 400 (Seward Laboratories Systems, Inc.) and 2 × 0.5 mL of the homogenate from each sample was aliquoted into Lysing Matrix E tubes (MP Biomedicals, LLC, Solon, OH) and stored at -80 °C until DNA extraction was performed. For city water/wastewater samples, additional 400 mL was filtered as described above and the filter was placed in Lysing Matrix E tube (MP Biomedical LLC).

### *Salmonella* isolation

As a pre-enrichment step, the stomached homogenates remained in the filtered stomacher bags and incubated overnight at 35 °C. Two different enrichments broths were used to isolate *Salmonella* spp. from these environmental samples: Tetrathionate (TT; Becton–Dickinson, Sparks, MD) broth and Rappaport–Vassiliadis (RV; Becton Dickinson) media. After overnight incubation at 42 °C in both enrichment broths, one loopful from each enrichment broth was spread on two different differential media: Brilliant Green Sulfa with novobiocin (BGS; Becton Dickinson) agar and xylose lysine tergitol-4 (XLT-4; Becton Dickinson) agar. These plates were incubated overnight at 35 °C, and on each plate, three *Salmonella*–like colonies per subsample were picked and confirmed using triple sugar iron agar (TSI; Becton–Dickinson) and lysine iron agar fermentation (LIA; Becton–Dickinson) using an incubation period of 18 to 24 h at 35 °C. Final confirmation of suspect TSI/LIA isolates was performed using *Salmonella* polyvalent O antiserum agglutination (Becton–Dickinson), using manufacturer’s specifications.

### DNA extraction and 16S rRNA sequence analysis

Genomic DNA was extracted from all samples according to a semi-automated hybrid DNA extraction protocol previously described [16]. This method was a combination of a mechanical method using the FastDNA Spin Kit for Feces (MP Biomedicals) and an enzymatic method based on the QIAamp DNA Stool Mini Kit (Qiagen, Valencia, CA). DNA purification was performed using the DNA Stool – Human Stool – Pathogen Detection Protocol of the QIAcube Robotic Workstation. After purification, DNA extracts were quantified using an Invitrogen Qubit 4 Fluorometer and 1 × dsDNA High Sensitivity Assay Kit (ThermoFisher Scientific, Waltham, MA, USA). All 16S rRNA Illumina-tag PCR reactions were performed on DNA extracts per the Earth Microbiome Project’s protocol [17]. Negative controls (PCR grade nuclease-free water) were processed in parallel with the samples for PCR amplification. PCR products were visualized on a 2% agarose E-Gel with ethidium bromide (Thermo Fisher Scientific) for bands at ~ 400 bp. PCR products (including any negatives that showed amplification) were pooled and gel purified on a 2% agarose gel using the QIAquick Gel Purification Kit (Qiagen, Frederick, Maryland, USA). Before sequencing, the purified pool was quality checked using an Agilent 2100 BioAnalyzer and Agilent DNA High Sensitivity DNA kit (Agilent Technologies, Santa Clara, California, USA). The concentration of all genomic DNA samples was between 1 and 10 ng/uL. The purified pool was stored at − 20 °C and then sequenced by Wright Labs (Huntingdon, PA, USA) using an Illumina MiSeq v2 chemistry with paired-end 250 base pair reads. The sequence data is available under SRA accession number PRJNA1103390.

Raw sequence reads obtained from the Illumina MiSeq were processed in *R* v4.3.0 [18] using the *DADA2* package v1.18 [19]. Only reads with a maximum number of expected errors lower than or equal to 2 were retained. In addition, reads were truncated where the phred quality score dropped below 30. Chimeras were identified and removed using the consensus method and the remaining reads were annotated to the SILVA database release 138 with a minimum bootstrap threshold of 50 [20]. Amplicon sequence variants (ASVs) with less than 5 sequences in total and ASVs with ambiguous annotations (Chloroplasts, Mitochondria) were removed from the dataset. Finally, samples with less than 5,000 sequences were removed. The average sequence depth per sample was 29,289.54, ranging from 6709 to 54,602 sequences. One sample was removed from the analysis due to lack of amplification.

In-depth microbial community analysis was performed in the R environment. Alpha diversity indices were calculated with a dataset rarefied to the smallest sample size using the *Phyloseq* package v1.44 [21]. Values of alpha diversity indices were checked for normal distribution by visually assessing qqplots and histograms and by calculating the Shapiro–Wilk normality test (*p* = 0.05). The groups that were not normally distributed were compared using the Wilcoxon Signed Rank test. A principal coordinate analysis (PCoA) based on Bray–Curtis distances was performed with the *Ampvis2* package v2.2.8 to calculate changes in microbial beta diversity [22]. In addition, a permutational multivariate analysis of variance (PERMANOVA) was performed to assess the influence of experimental factors on the microbial community using the *vegan* package v2.6.4 [23]. Prior to this analysis, ASV’s with less than 0.1% relative abundance in any sample have been removed.

ASVs were considered part of the core microbiota with a relative abundance cutoff above 0.01% and a prevalence cutoff above 80% of the samples for a given hatchery area or sample type. The Venn diagram of core ASVs was created with VennDiagram package [24]. Differences of individual ASVs between *Salmonella* positive and negative samples and correlations of ASVs and *Salmonella*-associated sequences were computed using MaAsLin2 v1.14.1 [25]. Only associations for ASVs with a minimum prevalence of 10% and a minimum relative abundance of 1% were calculated. Benjamini–Hochberg procedure was applied as a correction method for computing the q-values. Microbial source tracking was achieved with the software SourceTracker v1.0.0 and default parameters [26]. Samples taken from the facility environment were assigned as sources, whereas egg samples were assigned as sinks.

## Results and discussion

### Microbiota diversity metrics

Sample type category and hatchery area both significantly impacted the diversity of the microbiota within this study (Table 1). Microbiota from floor drain swabs (Water category) had significantly higher richness (*p* ≤ 0.0059), diversity (*p* ≤ 0.0034), and evenness (*p* ≤ 0.0084) estimates than the Egg and Air category microbiota. Generally, the lowest numerical estimates were found in the Air samples, although they were never statistically different than the Egg microbiota estimates. All three α-diversity estimates tended to decrease from the pre-hatch to post-hatch areas, with the Egg Inventory (EI) and Pre-*in ovo* (PrI) areas having significantly higher richness (*p* < 0.0001), diversity (*p* ≤ 0.0025), and evenness (*p* ≤ 0.0366) estimates than the Post-*in ovo* (PoI), Processing (PR), and Transport (T) areas, with the exception of the richness estimates between the PrI and T areas and the evenness estimates between the EI and PR areas (*p* = 0.0960 and 0.0633, respectively).

**Table 1:** Alpha diversity metrics for hatchery microbiota separated by sample type category and hatchery area

	Richness (Observed OTUs)	Diversity (Shannon)	Evenness (Equitability)
Sample Type Category^1^			
Air (N = 40)	357.82^B^	3.45^B^	0.5906^BC^
Egg (N = 40)	421.75^B^	3.53^B^	0.5808^C^
Water (N = 32)	534.81^A^	4.19^A^	0.6700^A^
FAC (N = 72)	396.31^B^	3.77^AB^	0.6344^AB^
Hatchery Area^1,2^			
EI (N = 32)	564.25^A^	4.26^A^	0.6741^A^
PrI (N = 32)	497.03^AB^	4.15^A^	0.6707^AB^
PoI (N = 24)	312.04^DE^	3.35^B^	0.5848^C^
PR (N = 32)	305.09^E^	3.44^B^	0.604^AC^
T (N = 24)	412.16^BC^	3.23^B^	0.5295^C^
FAC (N = 40)	388.44^CD^	3.54^B^	0.6028^BC^

^1^ Superscript letters indicate significantly different prevalence values based on Kruskal–Wallis analyses using the Wilcoxon post-test at a significance level of *p* < 0.05. Sample Type Category and Hatchery Areas were analyzed separately

^2^ EI = Egg inventory, PrI = Pre-in ovo incubation, PoI = Post-in ovo incubation, PR = Chick processing, T = Chick transport, FAC = Facility

**Fig. 2:**
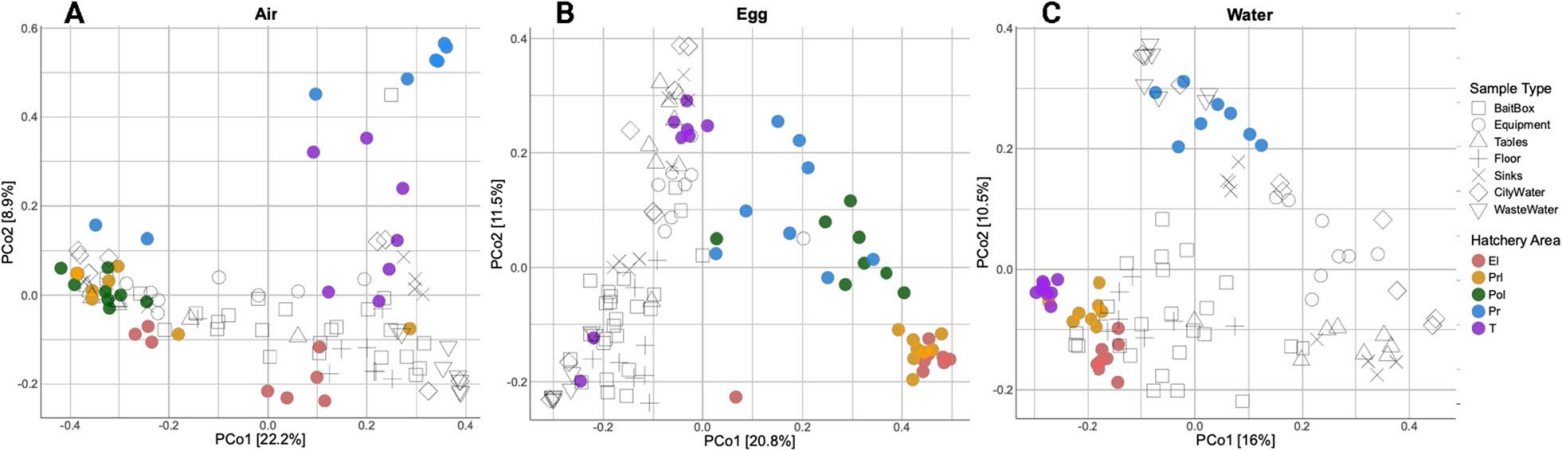
Beta Diversity PCoA plots for the **A** Air, **B** Egg, **C** Water samples. The different areas of the hatchery are color coded solid circles, while the open symbols and crosses represent different sample type categories within the facility-related samples

Like α-diversity, both sample type category and hatchery area significantly impacted the β-diversity of the recovered microbiota (*p* = 0.0002 and 0.0002, respectively). PCoA plots (Fig. 2) revealed a transition of microbiota from the EI (Red) to T (Purple) areas of the hatchery for the Air (Fig. 2A), Egg (Fig. 2B), and Water (Fig. 2C) category related samples. While the facility-related samples taken throughout the study (swabs of bait boxes, processing equipment, floors, sinks, breakroom tables, as well as filtration of the incoming city water and outgoing wastewater) displayed significant differences in the α-diversity metrics compared to the different sample type categories and hatchery areas (Table 1), these associations are more clearly demonstrated within these PCoA plots. The Air and Water category microbiota tended to shift away from the facility-related microbiota by the time the transport truck samples were taken (T; Fig. 2A and C, respectively), although these two types of microbiota did cluster with different groups of facility-related samples in different areas of the hatchery. For example, the floor drain microbiota (Water; Fig. 2C) clustered with or near the floor-associated facility samples. Specifically, the EI, PrI, and T samples clustered near the floor ( +) and bait box (□) swabs, while the PR samples (blue dots) clustered with the wastewater (∇) samples. Since washing of the PR area and equipment largely generated the wastewater that was collected, this shift in floor drain microbiota to emulate the wastewater microbiota is not surprising.

The Egg category microbiota shifted towards the facility-related microbiota in each successive hatchery area (Fig. 2B). In fact, the chick pad feces samples from the T area clustered directly with the facility-related microbiota, potentially indicating the influence of the hatchery facility on shaping the egg-related microbiota by the time the chicks are transported to the live production farms. The first few days post-hatch are crucial in the development of mature gut microbiota in broiler chicken, indicating that such a stable microbiota is already being established in the hatchery [27]. There is evidence that maternal oviduct microbiota influence the gut microbiome of the offspring [28], young chicks also encounter bacterial communities found on the eggshell and the nest environment [29]. These findings highlight the potential influence of the hatchery environment in shaping the gut microbiota in young hatchlings.

### Hatchery microbiota taxa composition

**Fig. 3:**
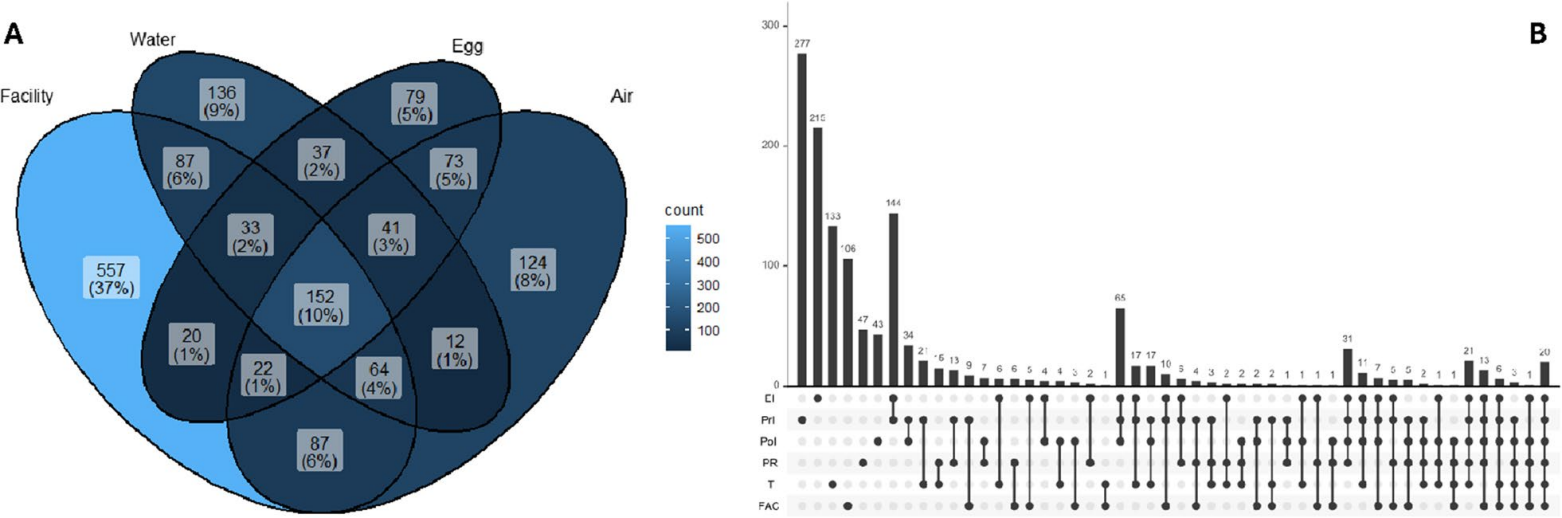
Venn diagrams representing the different combinations of core ASVs present in different sample combinations. **A** Combinations based on four different sample type categories containing the number of ASVs with the percentage of total core ASVs in parentheses. **B** Combinations based on the six different hatchery areas (EI = Egg inventory, PrI = Pre-in ovo Incubation, PoI = Post-in ovo incubation, PR = Chick processing, T = Transport, FAC = General facility). The histogram shows the number of core ASVs for each area combination defined below the x axis (black circle = included in combination, white circle = excluded from combination)

The core microbiotas (ASVs present in at least 80% of all samples for a given sample type or hatchery area) were determined, resulting in 1523 total ASVs among the core microbiota of at least one sample type or hatchery area. The Venn diagram (Fig. 3A) shows the number of ASVs present in every sample type. Over half of all core ASVs were unique to a single sample type category (8%, 5%, 9%, and 37% for Air, Egg, Water, and FAC, respectively), although nearly two-thirds were unique to the FAC samples. This was unsurprising since the FAC category encompasses several different environmental swabs and water samples. When looking at the phyla-level distribution of taxa unique to the three main sample type categories, ASVs related to Firmicutes and Bacteroidota were up to two times greater, and the ASVs related to Actinobacteriota and Proteobacteria were over two times lower for the core Egg microbiota compared to the core Air and Water microbiota (Table 2). Unique Deinococcota ASVs were only present in the Water samples. There were 152 ASVs (10%) present in the core microbiota of all sample type categories within the hatchery (Supplemental Table S2).

**Table 2:** Phyla-level distribution of unique Core ASVs according to sample type category and hatchery area

	Actinobacteriota	Bacteroidota	Deinococcota	Firmicutes	Proteobacteria
Sample type category^1^					
Air (124 ASVs)	22 (17.74%)	29 (23.39%)	0 (0.00%)	27 (21.77%)	38 (30.65%)
Egg (79 ASVs)	5 (6.33%)	27 (34.18%)	0 (0.00%)	34 (43.04%)	9 (11.39%)
Water (136 ASVs)	28 (20.59%)	22 (16.18%)	13 (9.56%)	30 (22.06%)	35 (25.74%)
Hatchery Area^1,2^					
EI (215 ASVs)	36 (16.67%)	59 (27.31%)	0 (0.00%)	71 (32.87%)	37 (17.13%)
PrI (277 ASVs)	41 (14.59%)	56 (19.93%)	8 (2.85%)	58 (20.64%)	97 (34.52%)
PoI (43 ASVs)	3 (6.67%)	10 (22.22%)	1 (2.22%)	3 (6.67%)	27 (60.00%)
PR (47 ASVs)	16 (32.65%)	4 (8.16%)	1 (2.04%)	7 (14.28%)	20 (40.82%)
T (133 ASVs)	26 (19.55%)	11 (8.27%)	28 (21.05%)	4 (3.01%)	57 (42.86%)

^1^ Value represent the total number of ASVs unique to that phyla for that sample type category or hatchery area, while the number in parentheses represents the percent of all unique ASVs for that phyla for that sample type category or hatchery area

^2^ EI = Egg inventory, PrI = Pre-in ovo incubation, PoI = Post-in ovo incubation, PR = Chick processing, T = Chick transport

When focusing on the area of the hatchery facility, 17.6% of the ASVs (268/1523) did not comprise the core microbiota of any specific area, but similar to the sample type category comparisons above, around 50% of the ASVs were unique to a single area (14%, 18%, 3%, 3%, 9%, and 7% for the EI, PrI, PoI, PR, T, and FAC areas, respectively; Fig. 3B). The phyla-level distribution of unique core ASVs (Table 2) showed a reduction in unique Bacteroidota and Firmicutes ASVs post-hatch (PoI, PR, T), with a concomitant increase in unique Proteobacteria ASVs (Table 2). Unique Deinococcota ASVs were enriched in the microbiota from the swabs of the transport trucks (T).

Various studies indicated an appreciable role of eggshell microbiota in shaping the microbial communities in the jejunum and ileum of young broilers [28, 30]. The composition of eggshell microbiota is greatly influenced by factors such as the laying environment (nest or floor) and the presence of visible dirt and feces on it [30]. Fertile eggs undergo extensive disinfection to eliminate pathogenic bacteria, with variations in disinfection processes contributing significantly to differences in eggshell microbial communities across hatcheries [30]. The core microbiota found on the eggshell on this study aligns with other studies results as the eggshell microbiota was mainly dominated with Firmicutes, Proteobacteria, and Actinobacteriota [2, 30, 32].

The presence of *Deinococcus* ASVs was unexpected. *Deinococcus* are a highly resistant bacteria that can survive ionizing and ultraviolet radiations. *Deinococcocus* spp. have gained considerable interest from industries, mainly due to their bioremediation capabilities and potential applications in nuclear waste management [33, 34]. While *Deinococcocus* do not typically cause human or animal diseases [35], their presence in the floor drain/transport truck underfloor reservoir swab samples may be indicative of their resilience towards the hygiene and disinfection processes used in the hatchery. Therefore, if future studies show *Deinococcus* spp. are prevalent within commercial hatcheries, they may act as a sentinel target to assess hatchery disinfection efficacy, as well as an indicator for other desiccation/disinfection resistant microbes to survive cleaning processes.

**Fig. 4:**
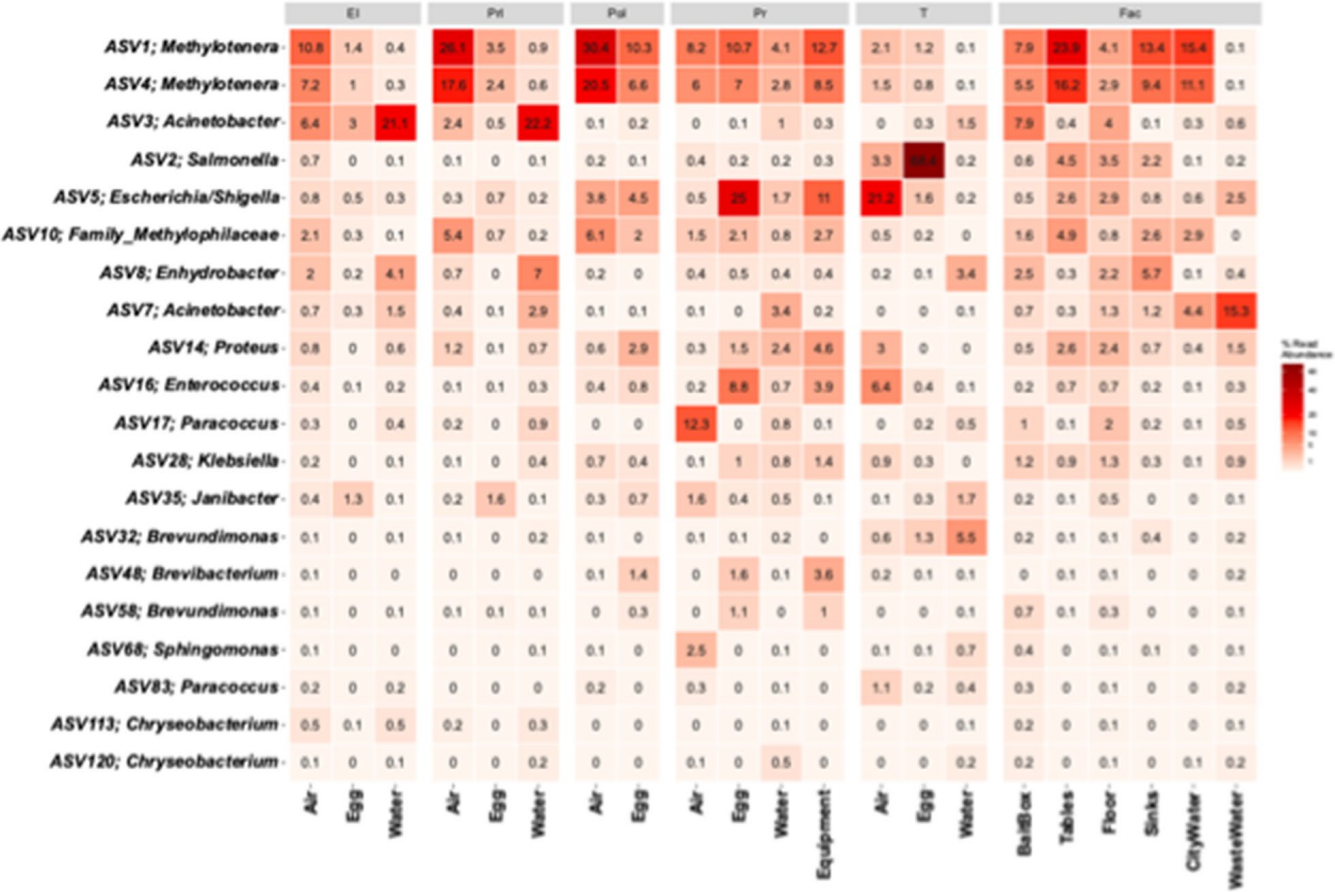
Relative abundance heatmap of the 20 core ASVs found in all sample type categories and hatchery areas (Core20). Text above the heatmap defines the different hatchery areas (EI = Egg inventory, PrI = Pre-in ovo incubation, PoI = Post-in ovo incubation, PR = Chick processing, T = Transport, FAC = General facility) while the text below defines the sample type category. The Core20 ASVs, preceded by the ASV number, are listed to the left of the heatmap, and the relative abundances for each ASV are presented within each cell of the heatmap. Any ASVs showing 0% indicates that their relative abundance is between 0.01 and 0.049%. The higher the relative abundance, the darker red the cell is shaded

It was observed that 20 ASVs (**Core20**) were part of the core microbiota of all sample type categories and all hatchery areas (Table 3), so given their ubiquity throughout the hatchery, they were investigated further. The Core20 are dominated by γ− and α– Proteobacteria (10 and 5 ASVs, respectively), with the remainder aligned with Actinobacteria (2), Bacteroides (2), and Bacilli (1). The distribution of the Core20 ASVs within the hatchery was sample type category and area dependent (Fig. 4). Two of the four most abundant ASVs within the hatchery microbiota were *Methylotenera* (ASV 1 and 4), and they were enriched in the Air microbiota throughout the facility, specifically in the pre-hatch/hatch areas. These two ASVs were also present in the FAC samples, specifically dominating the Table, Sink, and incoming City Water microbiota. The Water category microbiota were dominated in the pre-hatch areas by ASV3 *Acinetobacter*, representing 21.1% and 22.2% of the EI and PrI Water microbiota. Unlike the Water category samples, Core20 ASVs in the Egg microbiota in the pre-hatch areas (EI, PrI, PoI) were more uniformly distributed, but were dominated by very few ASVs in the hatch/post-hatch areas. ASV5 *Escherichia/Shigella*, ASV1 *Methylotenera*, and ASV16 *Enterococcus* represented 25%, and 10.7% and 8.8% of the PR area total eggshell/fluff microbiota, while the chick pad feces microbiota (T, Egg) were dominated by ASV2 *Salmonella* (68.4%).

**Table 3:** Taxonomic classification of the 20 ASVs (Core20) present in the core microbiota of all sample type categories and hatchery areas

ASV	Phylum	Class	Order	Family	Genus^1^	Species^1^
1	Proteobacteria	Gammaproteobacteria	Burkholderiales	Methylophilaceae	*Methylotenera*	
2	Proteobacteria	Gammaproteobacteria	Enterobacterales	Enterobacteriaceae	*Salmonella*	
3	Proteobacteria	Gammaproteobacteria	Pseudomonadales	Moraxellaceae	*Acinetobacter*	*lwoffii*
4	Proteobacteria	Gammaproteobacteria	Burkholderiales	Methylophilaceae	*Methylotenera*	
5	Proteobacteria	Gammaproteobacteria	Enterobacterales	Enterobacteriaceae	*Escherichia/Shigella*	
7	Proteobacteria	Gammaproteobacteria	Pseudomonadales	Moraxellaceae	*Acinetobacter*	*baumannii/bouvetii/haemolyticus/johnsonii/* *junii/ lwoffii/oleivorans/oryzae/schindleri*
8	Proteobacteria	Gammaproteobacteria	Pseudomonadales	Moraxellaceae	*Enhydrobacter*	
10	Proteobacteria	Gammaproteobacteria	Burkholderiales	Methylophilaceae		
14	Proteobacteria	Gammaproteobacteria	Enterobacterales	Morganellaceae	*Proteus*	
16	Firmicutes	Bacilli	Lactobacillales	Enterococcaceae	*Enterococcus*	
17	Proteobacteria	Alphaproteobacteria	Rhodobacterales	Rhodobacteraceae	*Paracoccus*	*carotinifaciens/gahaiensis/haeundaensis/* *hibiscisoli/ marcusii*
28	Proteobacteria	Gammaproteobacteria	Enterobacterales	Enterobacteriaceae	*Klebsiella*	
32	Proteobacteria	Alphaproteobacteria	Caulobacterales	Caulobacteraceae	*Brevundimonas*	*albigilva/nasdae/vesicularis*
35	Actinobacteriota	Actinobacteria	Micrococcales	Intrasporangiaceae	*Janibacter*	
48	Actinobacteriota	Actinobacteria	Micrococcales	Brevibacteriaceae	*Brevibacterium*	*luteolum*
58	Proteobacteria	Alphaproteobacteria	Caulobacterales	Caulobacteraceae	*Brevundimonas*	*diminuta/naejangsanensis/vancanneytii*
68	Proteobacteria	Alphaproteobacteria	Sphingomonadales	Sphingomonadaceae	*Sphingomonas*	*desiccabilis/hankookensis/leidyi/panni*
83	Proteobacteria	Alphaproteobacteria	Rhodobacterales	Rhodobacteraceae	*Paracoccus*	*aestuarii/beibuensis/hibisci/marinus/* *rhizosphaerae/ siganidrum/zhejiangensis*
113	Bacteroidota	Bacteroidia	Flavobacteriales	Weeksellaceae	*Chryseobacterium*	
120	Bacteroidota	Bacteroidia	Flavobacteriales	Weeksellaceae	*Chryseobacterium*	*taihuense*

^1^ A blank cell indicates that phylogeny could not be determined on this taxonomic level for this ASV

Bacterial communities found in commercial hatcheries are still poorly characterized, despite being the first bacterial communities encountered by the young hatchlings. Most data on the microbial communities in a hatchery stems from culture-based research conducted to track pathogens such as *Salmonella* and *E*. *coli* [36–38]. In our study, we noted a significant presence of *Methylotenera* ASVs (ASV1 and 4) in various hatchery areas. *Methylotenera* spp. are Gram-negative bacteria that use methylamine as their main energy source [39]. Methylamine, a colorless gas and a derivative of ammonia, is included in fungicides and active cleaning agents. The presence of methylamine in the hatchery resulting from the sanitary procedures might potentially explain the high abundance of *Methylotenera* throughout the different sample type categories and areas (Fig. 4).

### Ecology of *salmonella* within hatchery microbiota

**Fig. 5:**
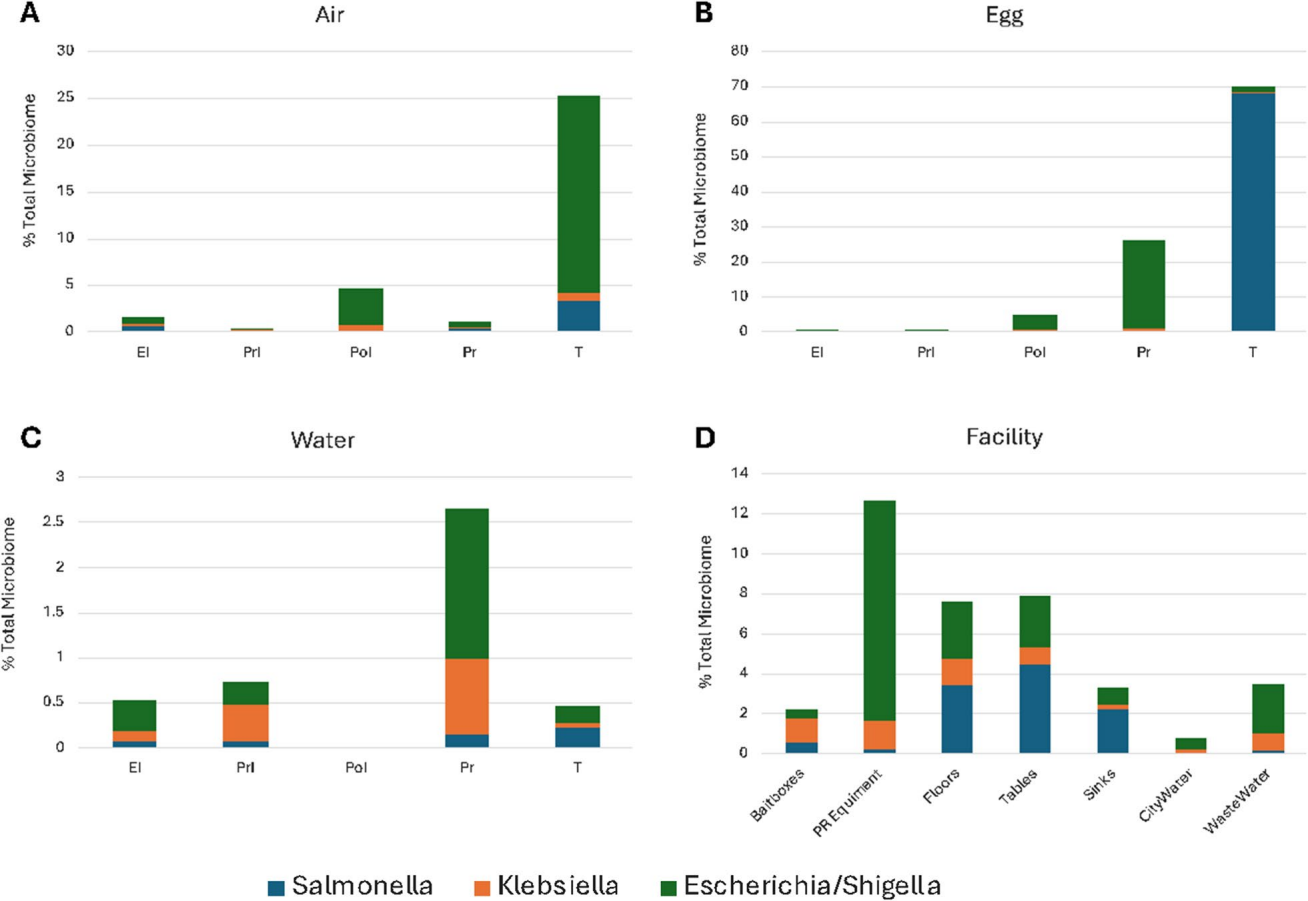
Relative abundance of Core20 Enterobacteriaceae ASVs in the **A** Air, **B** Egg, **C** Water, **D** Facility microbiota across the different areas of the hatchery

Considering *Salmonella* not only represented one of the Core20 ASVs, it was the second most abundant ASV across all microbiota within the entire hatchery dataset, and its food safety implications, the potential ecology of this ASV throughout the facility was further explored. The Core20 contained three ASVs classified as Enterobacteriaceae, which potentially fill a similar niche within the hatchery microbiota: ASV2 *Salmonella,* ASV5 *Escherichia/Shigella*, and ASV28 *Klebsiella*. When considering the distribution of these ASVs within the different hatchery microbiota, in the pre-hatch (EI, PrI, PoI) and hatch (PR) areas of the hatchery ASV5 *Escherichia/Shigella* comprised 45–80%, 88–95%, and 5–65% of the Core20 Enterobacteriaceae niche of the Air (Fig. 5A), Egg (Fig. 5B), and Water (Fig. 5C) microbiota, respectively. ASV28 *Klebsiella* was a minor constituent (< 1% of the total microbiota) in the Air and Egg microbiota, but they represented 20–55% Core20 Enterobacteriaceae niche in these prehatch and hatch areas of the Water microbiota. ASV2 *Salmonella* averaged a very small percentage of the Air, Egg, and Water microbiota in the pre-hatch and hatch areas (0.34%, 0.10%, and 0.10%, respectively), but in the Transport area the relative abundance increased 2–tenfold in the Water and Air samples, and 680 fold in the Egg samples, representing over two-thirds of all ASVs in the chick pad feces microbiota (Fig. 5B). ASV2 *Salmonella* also was the dominant ASV in the Core20 Enterobacteriaceae niche for the facility swabs of the high traffic floor areas, breakroom tables, and bathroom sinks (Fig. 5D).

*Salmonella* have been previously isolated from different areas and sample types in commercial broiler hatcheries [40] with commercial hatcheries being identified as a major source of *Salmonella*-related microbial risk of poultry products [4]. This aligns with a *Salmonella* ASV as one of the more prevalent Core20 taxa (Table 3). In our study, the interplay between three Enterobacteriaceae ASVs (*Salmonella, E. coli/Shigella, Klebsiella*) elucidates the niche competition between bacteria within any environment. It has been shown that *E. coli* can enhance the gut resistance against *Salmonella* Typhimurium by competing for the carbon source galacitol [41]. Similarly, Osbelt et al. (2021) demonstrated how limiting the carbohydrate sources can encourage interspecies competition for limited resources among the Enterobacteriaceae family [42]. Therefore, the proliferation of *Salmonella* in the transport area may be partially attributed to the decrease in the presence of Enterobacteriaceae family, which gave *Salmonella* a perfect niche to proliferate.

**Fig. 6:**
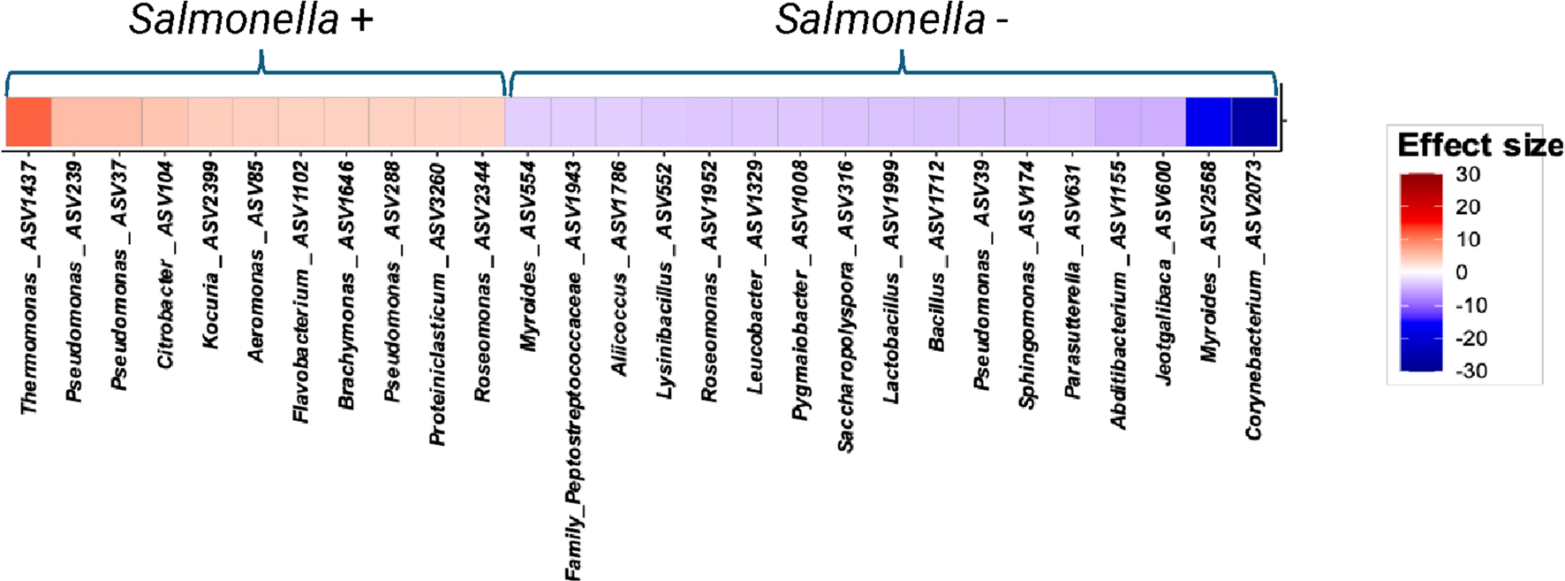
Differential ASV abundance based on cultural *Salmonella* status of hatchery samples based on the MaAsLin2 v1.14.1 algorithm. ASVs significantly enriched in *Salmonella* positive samples are shaded in red, while ASVs significantly enriched in *Salmonella* negative samples are shaded in blue, with greater significance indicated by intensity of the shading

Since *Salmonella* was isolated culturally as a separate part of this study, the differential abundance of ASVs based on the culturable *Salmonella* status ( ±) of each sample was investigated using MaAsLin2 v1.14.1 (Fig. 6). Twenty-six percent of all samples were *Salmonella* positive (48/184), so it was not surprising that there was no significant association between culturable *Salmonella* status and ASV2 *Salmonella* (*p* = 0.3724) given that the Core20 ASVs were present in at least 80% of the samples. Eleven ASVs were found to be significantly enriched in *Salmonella* positive samples (red shading), while 17 ASVs were significantly enriched in the *Salmonella* negative samples (blue shading). Proteobacteria ASVs comprised 8 of the 11 ASVs enriched in the *Salmonella* positive samples, including the strongest associated ASV (ASV1437 *Thermomonas*). The ASVs enriched in the *Salmonella* negative samples were distributed across five different phyla, although the Firmicutes ASVs (n = 7) were more than double those specific to any other phyla (3 for the Actinobacteria, Bacteroidota, and Proteobacteria). When *Salmonella* status was based on the presence of *Salmonella* ASVs in each sample’s microbiota (not culture-based), the enrichment of Proteobacteria ASVs in samples with a positive *Salmonella* status was evident (Supplemental Fig. S1). These results indicate that the conditions that are impacting the prevalence of *Salmonella* are potentially niche-related, since other Proteobacteria ASVs are concurrently enriched in the positive samples. Since the presence of closely related bacterial species increases the likelihood of a new bacterial species entering the same niche [43], certain environmental conditions may confer a fitness advantage to all members of a phylogenetic group, which could possibly explain the enrichment of Proteobacteria ASVs in *Salmonella* positive samples.

**Fig. 7:**
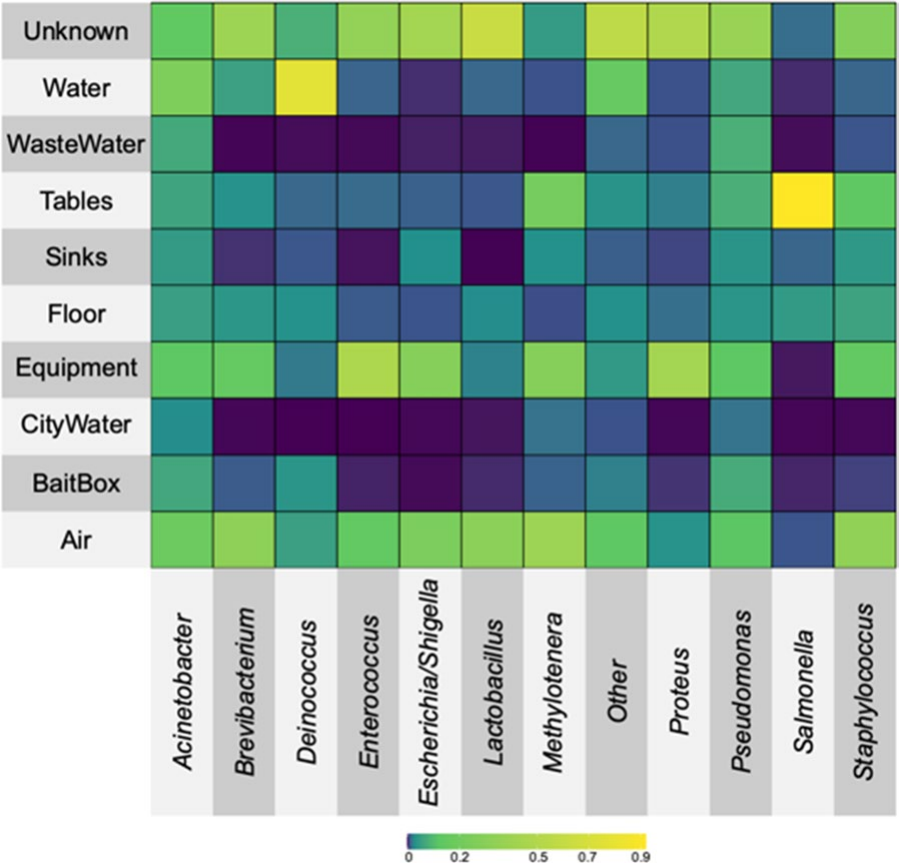
Identification of potential sources of the dominant core Egg ASVs within the hatchery facility based on the SourceTracker 1.0.0 algorithm. Dominant (> 1% of total core Egg microbiota) ASVs are listed along the bottom of the heatmap, while the potential facility sources are listed to the left. The color shading ranges from dark green (no association) to yellow (strongest association)

Since ASV2 *Salmonella* was 0.25%—4.46% of the total microbiota of the various facility swab samples (Fig. 5D), a microbial source tracking algorithm (SourceTracker v1.0.0) was used to determine the most likely sources of the most abundant ASVs (ASVs representing > 1% of all core Egg microbiota) in the Egg category samples (Fig. 7). While the two sources for the top core Egg ASVs overall were either air-related samples or unknown (not attributable to any specific sample type) (lighter green to greenish-yellow colors), the strongest association (represented by the bright yellow shading) was between ASV2 *Salmonella* and the swabs from the breakroom tables. These findings do not indicate directionality of *Salmonella* movement within the hatchery; it only suggests the breakrooms as a potential reservoir for *Salmonella* associated with the chick pad feces. This potential of the human-related surfaces as *Salmonella* reservoirs within the poultry-associated facilities or food service-related areas have been previously observed from disinfected surface equipment in poultry processing plants [44, 45], as well as cutting boards and dish clothes in restaurants [46]. Therefore, this analysis, in combination with the trend of the Egg microbiota community structure becoming more similar to the facility microbiota as the eggs move from the pre-hatch to post-hatch areas (Fig. 2B), potentially indicate the facility, and possibly the human workers, as *Salmonella* reservoirs within the hatchery. These findings highlight prospective critical control points for mitigation efforts to reduce *Salmonella* entering the live production houses that required further investigation.

## Conclusion

Within this studies’ commercial hatchery, microbiota were significantly affected by the type of sample being analyzed, as well as the area of the hatchery from which the sample was collected. While each sample type category and hatchery area possessed unique ASVs, 20 ASVs were present in the core microbiota of all sample type categories and hatchery areas (Core20), with ASVs related to Proteobacteria representing 75% of the Core20. Three ASVs related to Enterobacteriaceae (*Salmonella, E. coli/Shigella, Klebsiella*) were part of the Core20, and their distributions were sample type and hatchery area dependent, with *Salmonella* dominating the microbiota of the Egg samples in the Transport area. Further investigations of the *Salmonella* ASV showed an association of this ASV within the Egg microbiota at Transport with the breakroom tables, potentially indicating a cross-contamination reservoir for *Salmonella* within the hatchery. These results highlight the utility of microbiota analyses to better understand the microbial ecology of the hatchery environment, as well as identifying potential sampling targets (chick pad feces in the transport area) or critical control points (breakroom tables) within the facility for hatchery managers to mitigate *Salmonella* loads from entering the live production houses.

### Supplementary Information

Additional file 1

Additional file 2

Additional file 3

Additional file 4

Additional file 5

## Data Availability

The sequence data that support the findings of this study have been reposted in the National Institute of Health (NIH)'s Sequence Read Archive (SRA) and is available under accession number PRJNA1103390.

## References

[1] Wales A, Davies R. Review of hatchery transmission of bacteria with focus on *Salmonella*, chick pathogens and antimicrobial resistance. Worlds Poult Sci J. 2020;76:517–36. 10.1080/00439339.2020.1789533

[2] Ballou AL, Ali RA, Mendoza MA, Ellis JC, Hassan HM, Croom WJ, Koci MD. Development of the chick microbiome: how early exposure influences future microbial diversity. Front Veterinary Sci. 2016;3:2. 10.3389/fvets.2016.00002 PMC4718982 26835461

[3] Rothrock MJ Jr, Locatelli A, Feye KM, Caudill AJ, Guard J, Hiett K, Ricke SC. A microbiomic analysis of a pasture-raised broiler flock elucidates foodborne pathogen ecology along the farm-to-fork continuum. Front Veterinary Sci. 2019;6:260. 10.3389/fvets.2019.00260 PMC6692657 31448296

[4] Wang J, Vaddu S, Bhumanapalli S, Mishra A, Applegate T, Singh M, Thippareddi H. A systematic review and meta-analysis of the sources of Salmonella in poultry production (pre-harvest) and their relative contributions to the microbial risk of poultry meat. Poult Sci. 2023;102: 102566. 36996513 10.1016/j.psj.2023.102566 PMC10074252

[5] U.S. Department of Agriculture-National Agricultural Statistics Service. 2024. Poultry: production and value 2023 summary. 2024. https://downloads.usda.library.cornell.edu/usda-esmis/files/m039k491c/b2775j31b/9k4213149/plva0424.pdf. Accessed 9 July 2024.

[6] Fa Mansour A, Zayed A. AA Basha O: contamination of the shell and internal content of table eggs with some pathogens during different storage periods. Assiut Vet Med J. 2015;61:8–15. 10.21608/avmj.2015.169765

[7] Bailey J, Buhr R, Cox N, Berrang M. Effect of hatching cabinet sanitation treatments on *Salmonella* cross-contamination and hatchability of broiler eggs. Poult Sci. 1996;75:191–6. 8833369 10.3382/ps.0750191

[8] Osman KM, Kappell AD, Elhadidy M, ElMougy F, El-Ghany WAA, Orabi A, Mubarak AS, Dawoud TM, Hemeg HA, Moussa IM. Poultry hatcheries as potential reservoirs for antimicrobial-resistant *Escherichia coli*: A risk to public health and food safety. Sci Rep. 2018;8:5859. 29643424 10.1038/s41598-018-23962-7 PMC5895583

[9] Messens W, Grijspeerdt K, Herman L. Eggshell penetration by *Salmonella*: a review. Worlds Poult Sci J. 2005;61:71–86. 10.1079/WPS200443

[10] Rezaee MS, Liebhart D, Hess C, Hess M, Paudel S. Bacterial infection in chicken embryos and consequences of yolk sac constitution for embryo survival. Vet Pathol. 2021;58(1):71–9. 33016240 10.1177/0300985820960127

[11] Cason J, Cox N, Bailey J. Transmission of *Salmonella* typhimurium during hatching of broiler chicks. Avian Dis. 1994;38:583–8. 7832712 10.2307/1592082

[12] Armour NK, Ferguson-Noel N. Evaluation of the egg transmission and pathogenicity of *Mycoplasma **gallisepticum* isolates genotyped as ts-11. Avian Pathol. 2015;44:296–304. 25925422 10.1080/03079457.2015.1044890

[13] Feye K, Thompson D, Rothrock M Jr, Kogut M, Ricke S. Poultry processing and the application of microbiome mapping. Poult Sci. 2020;99:678–88. 32029154 10.1016/j.psj.2019.12.019 PMC7587767

[14] Handley JA, Park SH, Kim SA, Ricke SC. Microbiome profiles of commercial broilers through evisceration and immersion chilling during poultry slaughter and the identification of potential indicator microorganisms. Front Microbiol. 2018;9:345. 29552001 10.3389/fmicb.2018.00345 PMC5841210

[15] Muyyarikkandy MS, Parzygnat J, Thakur S. Uncovering changes in microbiome profiles across commercial and backyard poultry farming systems. Microbiol Spectrum. 2023;11:e01682-e11623. 10.1128/spectrum.01682-23 PMC10580917 37607066

[16] Rothrock MJ Jr, Hiett KL, Gamble J, Caudill AC, Cicconi-Hogan KM, Caporaso JG. A hybrid DNA extraction method for the qualitative and quantitative assessment of bacterial communities from poultry production samples. JoVE (J Visual Exp). 2014;94:e52161. 10.3791/52161 PMC4396950 25548939

[17] Thompson LR, Sanders JG, McDonald D, Amir A, Ladau J, Locey KJ, Prill RJ, Tripathi A, Gibbons SM, Ackermann G. A communal catalogue reveals Earth’s multiscale microbial diversity. Nature. 2017;551:457–63. 29088705 10.1038/nature24621 PMC6192678

[18] Team RC.R Core Team R: A language and environment for statistical computing. *Foundation for Statistical Computing*,2020.

[19] Callahan BJ, McMurdie PJ, Rosen MJ, Han AW, Johnson AJA, Holmes SP. DADA2: high-resolution sample inference from Illumina amplicon data. Nat Methods. 2016;13:581–3. 27214047 10.1038/nmeth.3869 PMC4927377

[20] Quast C, Pruesse E, Yilmaz P, Gerken J, Schweer T, Yarza P, Peplies J, Glöckner FO. The SILVA ribosomal RNA gene database project: improved data processing and web-based tools. Nucl Acids Res. 2012;41:D590–6. 23193283 10.1093/nar/gks1219 PMC3531112

[21] McMurdie PJ, Holmes S. phyloseq: an R package for reproducible interactive analysis and graphics of microbiome census data. PLoS ONE. 2013;8: e61217. 23630581 10.1371/journal.pone.0061217 PMC3632530

[22] Andersen KS, Kirkegaard RH, Karst SM, Albertsen M: ampvis2: an R package to analyse and visualise 16S rRNA amplicon data. *BioRxiv* 2018:299537.

[23] Oksanen J, Blanchet F, Kindt R, Legendre P, Minchin P, OLHara R, Simpson G, Solymos P, Henry M, Stevens H: vegan: Community ecology package. R package version 2.0–10. CRAN 2013.

[24] Chen H, Boutros PC. VennDiagram: a package for the generation of highly-customizable Venn and Euler diagrams in R. BMC Bioinf. 2011;12:1–7. 10.1186/1471-2105-12-35 PMC3041657 21269502

[25] Mallick H, Rahnavard A, McIver LJ, Ma S, Zhang Y, Nguyen LH, Tickle TL, Weingart G, Ren B, Schwager EH. Multivariable association discovery in population-scale meta-omics studies. PLoS Comput Biol. 2021;17: e1009442. 34784344 10.1371/journal.pcbi.1009442 PMC8714082

[26] Knights D, Kuczynski J, Charlson ES, Zaneveld J, Mozer MC, Collman RG, Bushman FD, Knight R, Kelley ST. Bayesian community-wide culture-independent microbial source tracking. Nat Methods. 2011;8:761–3. 21765408 10.1038/nmeth.1650 PMC3791591

[27] Maki JJ, Bobeck EA, Sylte MJ, Looft T. Eggshell and environmental bacteria contribute to the intestinal microbiota of growing chickens. J Animal Sci Biotechnol. 2020;11:60. 10.1186/s40104-020-00459-w PMC7288515 32537141

[28] Ding J, Dai R, Yang L, He C, Xu K, Liu S, Zhao W, Xiao L, Luo L, Zhang Y. Inheritance and establishment of gut microbiota in chickens. Front Microbiol. 1967;2017:8. 10.3389/fmicb.2017.01967 PMC5641346 29067020

[29] Li X, Bi R, Xiao K, Roy A, Zhang Z, Chen X, Peng J, Wang R, Yang R, Shen X. Hen raising helps chicks establish gut microbiota in their early life and improve microbiota stability after H9N2 challenge. Microbiome. 2022;10:14. 35074015 10.1186/s40168-021-01200-z PMC8785444

[30] Maki JJ, Bobeck EA, Sylte MJ, Looft T. Eggshell and environmental bacteria contribute to the intestinal microbiota of growing chickens. J Animal Sci Biotechnol. 2020;11:1–17. 10.1186/s40104-020-00459-w PMC7288515 32537141

[31] Oliveira GdS. McManus C, Salgado CB, Dos Santos VM: Effects of sanitizers on microbiological control of hatching eggshells and poultry health during embryogenesis and early stages after hatching in the last decade. Animals. 2022;12:2826. 36290211 10.3390/ani12202826 PMC9597748

[32] Trudeau S, Thibodeau A, Côté JC, Gaucher ML, Fravalo P. Contribution of the broiler breeders’ fecal microbiota to the establishment of the eggshell microbiota. Front Microbiol. 2020;15(11):666. 10.3389/fmicb.2020.00666 PMC7176364 32351488

[33] Li S, Zhu Q, Luo J, Shu Y, Guo K, Xie J, He S. Application progress of deinococcus radiodurans in biological treatment of radioactive uranium-containing wastewater. Indian J Microbiol. 2021;61(4):417–26. 34744197 10.1007/s12088-021-00969-9 PMC8542025

[34] Chen A, Sherman MW, Chu C, Gonzalez N, Patel T, Contreras LM. Discovery and characterization of native Deinococcus radiodurans promoters for tunable gene expression. Appl Environ Microbiol. 2019;85(21):e01356-e1419. 31471304 10.1128/AEM.01356-19 PMC6803307

[35] Chen F, Zhang J, Ji HJ, Kim M-K, Kim KW, Choi J-I, Han SH, Lim S, Seo HS, Ahn KB. Deinococcus radiodurans exopolysaccharide inhibits Staphylococcus aureus biofilm formation. Front Microbiol. 2021;12: 712086. 35002990 10.3389/fmicb.2021.712086 PMC8739996

[36] Bailey J, Cox N, Berrang M. Hatchery-acquired *salmonellae* in broiler chicks. Poult Sci. 1994;73:1153–7. 7937477 10.3382/ps.0731153

[37] Oastler CE, Nichols C, Newton K, Cawthraw S, Gosling RJ, Martelli F, Wales AD, Davies RH. Observations on the distribution and control of *Salmonella* in commercial broiler hatcheries in Great Britain. Zoonoses Public Health. 2022;69:487–98. 35304827 10.1111/zph.12938 PMC9543921

[38] Shehata AA, Basiouni S, Elrazek AA, Sultan H, Tarabees R, Abd MS, Elsayed E, Talat S, Moharam I, Said A, Mohsen WA. Characterization of *Salmonella **enterica* isolated from poultry hatcheries and commercial broiler chickens. Pak Vet J. 2019;39(4):515–20. 10.29261/pakvetj/2019.033

[39] Kalyuzhnaya MG, Bowerman S, Lara JC, Lidstrom ME, Chistoserdova L. Methylotenera mobilis gen. nov., sp. nov., an obligately methylamine-utilizing bacterium within the family Methylophilaceae. Int J Syst Evolut Microbiol. 2006;56:2819–23. 10.1099/ijs.0.64191-0 17158982

[40] Willson N-L, Chousalkar K. Dominant *Salmonella* Serovars in Australian. Appl Environ Microbiol. 2023;89:e00627-e1623. 37466445 10.1128/aem.00627-23 PMC10467335

[41] Eberl C, Weiss AS, Jochum LM, Raj ACD, Ring D, Hussain S, Herp S, Meng C, Kleigrewe K, Gigl M. *E coli*. enhance colonization resistance against Salmonella Typhimurium by competing for galactitol, a context-dependent limiting carbon source. Cell Host Microbe. 2021;29(1680–1692):e1687. 10.1016/j.chom.2021.09.004 34610296

[42] Osbelt L, Wende M, Almási É, Derksen E, Muthukumarasamy U, Lesker TR, Galvez EJ, Pils MC, Schalk E, Chhatwal P. Klebsiella oxytoca causes colonization resistance against multidrug-resistant K pneumoniae in the gut via cooperative carbohydrate competition. Cell Host Microbe. 2021;29(1663–1679):e1667. 10.1016/j.chom.2021.09.003 34610293

[43] Stecher B, Chaffron S, Käppeli R, Hapfelmeier S, Freedrich S, Weber TC, Kirundi J, Suar M, McCoy KD, Von Mering C. Like will to like: abundances of closely related species can predict susceptibility to intestinal colonization by pathogenic and commensal bacteria. PLoS Pathog. 2010;6: e1000711. 20062525 10.1371/journal.ppat.1000711 PMC2796170

[44] Obe T, Nannapaneni R, Schilling W, Zhang L, McDaniel C, Kiess A. Prevalence of *Salmonella **enterica* on poultry processing equipment after completion of sanitization procedures. Poult Sci. 2020;99:4539–48. 32867998 10.1016/j.psj.2020.05.043 PMC7598133

[45] Zeng H, De Reu K, Gabriël S, Mattheus W, De Zutter L, Rasschaert G. *Salmonella* prevalence and persistence in industrialized poultry slaughterhouses. Poult Sci. 2021;100: 100991. 33610890 10.1016/j.psj.2021.01.014 PMC7905466

[46] Bolton DJ, Meally A, McDowell D, Blair IS. A survey for serotyping, antibiotic resistance profiling and PFGE characterization of and the potential multiplication of restaurant *Salmonella* isolates. J Appl Microbiol. 2007;103:1681–90. 17953579 10.1111/j.1365-2672.2007.03406.x

