# Supplementary material for: 16S amplicon-based microbiome biomapping of a commercial broiler hatchery
Source: Anim Microbiome. 2024 Aug 9;6:46. doi: 10.1186/s42523-024-00334-3 (PMC11312677; doi:10.1186/s42523-024-00334-3)
Supplement: Supplementary file 2 — Additional file 2 [file 42523_2024_334_MOESM2_ESM.docx]

Supplemental Table S1. Core ASVs for hatchery microbiomes separated by sample type category and hatchery area. Text in the first column in the first sheet defines sample types.

Supplemental Table S2. Core ASVs for hatchery microbiomes separated by hatchery area. Text in the first column in the second sheet defines the different hatchery areas (EI = Egg Inventory, PrI = Pre-in ovo Incubation, PoI = Post-in ovo Incubation, PR = Chick Processing, T = Transport, FAC = General Facility).

Supplemental Fig. S1. Differential ASV abundance based on microbiome Salmonella status of hatchery samples based on the MaAsLin2 v1.14.1 algorithm. ASVs significantly enriched in Salmonella positive samples are shaded in red, while ASVs significantly enriched in Salmonella negative samples are shaded in blue, with greater significance indicated by intensity of the shading.
